# Sleep Experiments

**Published:** 1914-05

**Authors:** 


Sleep Experiments. Claparede, Pieron and Legendre, *La Rif. Med.,* March 22, 1913, have found that dogs, kept from sleeping for ten days, die, but that if allowed to sleep a few days earlier, they recover. Cephalorhachidian fluid, injected from a fatigued dog into a normal animal, quickly produces sleep. This proves, what has long been known, that sleep is due to the accumulation of toxic products. Whether it would be justifiable to adopt the therapeutic hint is doubtful. On the whole, experiments of this sort rather strengthen the opponents of animal experimentation.

